# Human cytomegalovirus and Herpes Simplex type I virus can engage RNA polymerase I for transcription of immediate early genes

**DOI:** 10.18632/oncotarget.22106

**Published:** 2017-10-29

**Authors:** Ourania N. Kostopoulou, Vanessa Wilhelmi, Sina Raiss, Sharan Ananthaseshan, Mikael S. Lindström, Jiri Bartek, Cecilia Söderberg-Naucler

**Affiliations:** ^1^ Department of Medicine, Center for Molecular Medicine L8:03, Karolinska University Hospital, Stockholm, Sweden; ^2^ Department of Medical Biochemistry and Biophysics, Science For Life Laboratory, Division of Genome Biology, Karolinska Institute, Solna, Sweden

**Keywords:** cytomegalovirus, replication, RNA Polymerase I, Herpes Simplex type 1 virus, nucleolus

## Abstract

Human cytomegalovirus (HCMV) utilizes RNA polymerase II to transcribe viral genes and produce viral mRNAs. It can specifically target the nucleolus to facilitate viral transcription and translation. As RNA polymerase I (Pol I)-mediated transcription is active in the nucleolus, we investigated the role of Pol I, along with relative contributions of the human Pol II and Pol III, to early phases of viral transcription in HCMV infected cells, compared with Herpes Simplex Virus-1 (HSV-1) and Murine cytomegalovirus (MCMV). Inhibition of Pol I with siRNA or the Pol I inhibitors CX-5461 or Actinomycin D (5nM) resulted in significantly decreased IE and pp65 mRNA and protein levels in human fibroblasts at early times post infection. This initially delayed replication was compensated for later during the replication process, at which stage it didn’t significantly affect virus production. Pol I inhibition also reduced HSV-1 ICP0 and gB transcripts, suggesting that some herpesviruses engage Pol I for their early transcription. In contrast, inhibition of Pol I failed to affect MCMV transcription. Collectively, our results contribute to better understanding of the functional interplay between RNA Pol I-mediated nucleolar events and the Herpes viruses, particularly HCMV whose pathogenic impact ranges from congenital malformations and potentially deadly infections among immunosuppressed patients, up to HCMV’s emerging oncomodulatory role in human tumors.

## INTRODUCTION

Human cytomegalovirus (HCMV) is a beta herpesvirus that has infected 40% - 100% of the adult population worldwide [1]. Most infections are mild or even asymptomatic and occur in childhood. A primary infection is followed by life-long latency and persistence, mainly in myeloid lineage cells from where it can be reactivated [2, 3]. HCMV infects and replicates in a wide variety of cell types, including macrophages, dendritic cells, epithelial cells of glands and mucosal tissues, smooth muscle cells, fibroblasts, hepatocytes and vascular endothelial cells [4], and hence all bodily fluids and organs can transmit the virus. While HCMV is not considered to be pathogenic in healthy individuals with a normal immune system, the virus can cause life-threatening disease in immunocompromised patients such as organ and stem cell transplant patients and AIDS patients. Indeed, although ganciclovir has been available for treatment and prevention of HCMV infections for almost three decades, this virus still causes concerning morbidity and mortality in stem cell and organ transplant patients and in AIDS patients. It is also an important human pathogen to cause congenital infection with considerable risk of birth defects. Emerging evidence also suggests that HCMV is highly prevalent in breast, colon, and prostate cancer, rhabdomyosarcoma, hepatocellular cancer, salivary gland tumors, neuroblastoma and brain tumors (medulloblastoma and glioblastoma) [5-12]. This virus is not considered to be oncogenic per se, but rather promotes oncomodulatory functions leading to more aggressive cancer phenotypes and may thereby promote tumor progression. In such scenario, control of HCMV infections may affect the patients’ prognosis. In support of this statement, anti-viral treatment of patients with HCMV positive glioblastoma tumors indicate highly improved patient survival rates [13].

HCMV is the largest member of the herpesvirus family and consists of a linear double-stranded DNA genome of ∼240 kb that encodes from 170 [14] to 750 proteins [15]. Only about 50 of those proteins are believed to be essential for virus replication and production of new viral progeny. Instead, the vast majority of proteins aid the virus to interfere with cellular and immunological functions to enable it to coexist with its host [16]. HCMV also utilizes multiple strategies to exploit host functions for efficient virus production and its own spread and survival. Many of these are believed to confer oncomodulatory functions.

The virus life cycle is initiated after entry into the cells; the first genes expressed are the immediate early (IE) genes, producing IE proteins that serve as regulatory proteins acting as transcription factors to control both viral early and late gene expression as well as host gene expression. HCMV is known to utilize RNA polymerase II (Pol II) to transcribe the viral genes and accurate IE gene expression requires specific phosphorylation of the RNA Pol II CTD (carboxyl-terminal domain) early in infection [17]. The roles of the other two polymerases, Pol I and Pol III, in HCMV infection are unknown.

Many viruses including HCMV can specifically target the nucleolus to facilitate viral transcription and translation. The nucleolus is a membrane less nuclear organelle that assembles ribosomal subunits in eukaryotic cells [18, 19]. The ribosomal assembly process is triggered by activation of RNA polymerase I (Pol I)-mediated transcription and is regulated in a cell cycle-dependent manner in mammalian cells [20]. Mammalian cells contain several hundred copies of tandemly repeated rRNA genes (rDNA), which are transcribed in the nucleoli with high efficiency to meet the cell’s demand for ribosomes and protein synthesis. The Pol I transcription machinery consists of three main components: the Pol I enzyme, the TBP (TATA-binding protein) - TAF (TBP-associated factor) complex SL1 (selectivity factor 1) /TIF-IB (transcription initiation factor-IB) and the transactivator protein UBF (upstream binding factor) (reviewed in [21-23]). During tumorigenesis, the tightly regulated relationship between extracellular signalling and ribosome biosynthesis is disrupted, and cancer cells begin the excessive production of ribosomes necessary for the protein synthesis associated with uncontrolled cancer growth. rRNA is a major component of the ribosome and, as such, carcinogenesis requires an increase in its synthesis [24-26]. The CMV IE72 and pp65 proteins localize to the nucleoli during early infection, co-localize with the nucleolar protein fibrillarin and directly interact with nucleolin [27]. It has been proposed that HCMV induces rRNA synthesis and UBF transcription, as the nucleoli are enlarged in infected cells and change morphology. Upon specific inhibition of rRNA synthesis, both IE72 and pp65 exit the nucleolus resulting in a strong inhibition of HCMV infection [28-29]. HCMV also impacts the function of Rb, p53 and Myc, which would be predicted to interact with the Pol I machinery. However, despite such intriguing links, the effect of HCMV on rRNA transcription and the virus potential ability to utilize Pol I for its own transcription remains to be investigated.

The primary goal of the present study was to examine if Pol I inhibition affects the transcription of HCMV genes and/or virus production. In parallel, relative contributions of RNA Pol II and Pol III to HCMV early gene transcription were assessed as was the impact of Pol I interference in human cells infected by Herpes Simplex Virus-1 (HSV-1), and in mouse cells infected by the Murine cytomegalovirus (MCMV). Pol I inhibition was successfully achieved in two ways: using siRNA against Pol I or by treating cells with the specific Pol I inhibitor CX-5461 (a compound in clinical trials for oncology [30]) or low dose Actinomycin D (ActD). The results obtained from these experiments are presented below, followed by discussion of a potential biological significance of these findings for viral replication and (patho)-biology in relation to various cell types.

## RESULTS

### Inhibition of Pol I reduces transcription of HCMV immediate early (IE) and pp65 mRNA and protein expression in HCMV infected fibroblasts

**Figure 1:**
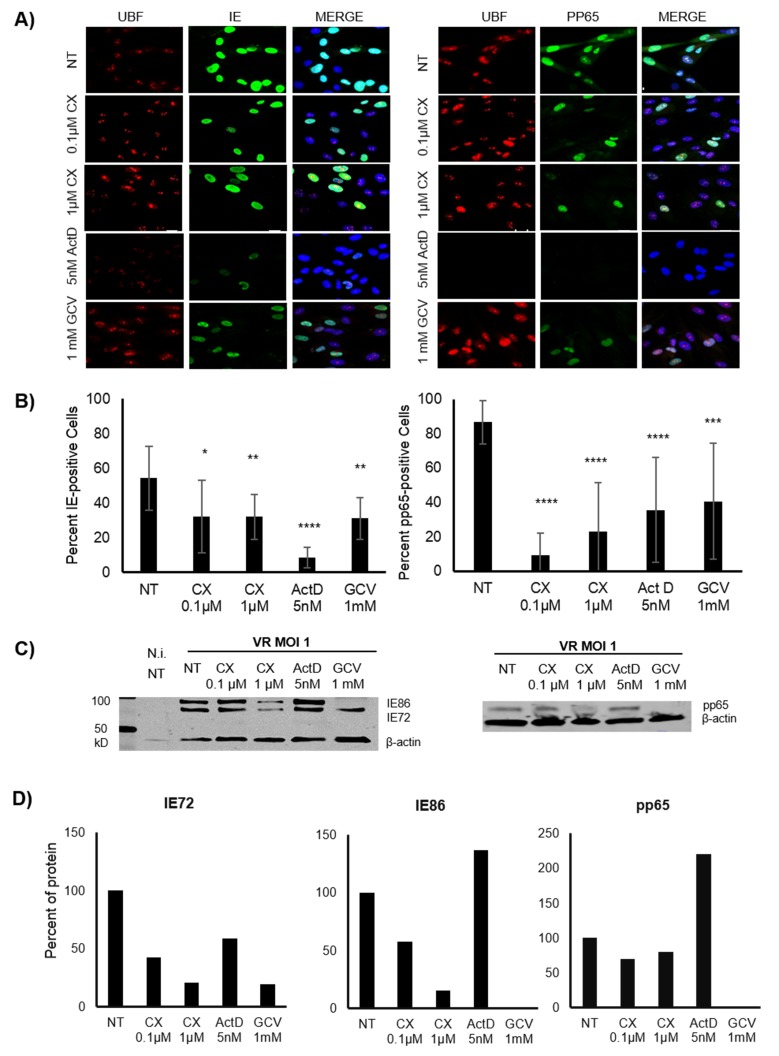
Effect of Pol I inhibition by CX-5461 on the HCMV immediate early (IE) and pp65 proteins in HCMV infected MRC5 cells **A.** Representative images of immunofluorescent staining (IF) for HCMV IE (green on the left column) and pp65 (green on the right column) proteins of pre-treated HCMV infected MRC5 cells. The cells were treated for 1.5h with 0.1μΜ, 1μΜ CX-5461, 5nM ActD or 1mM GCV and then HCMV (VR1814) infected for 48h. Actinomycin D (ActD) was used as control drug for Pol I inhibition and Ganciclovir (GCV) as antiviral control drug. UBF was stained as control for nucleolus. Nuclei were stained with DAPI (blue). **B.** Quantification of the percent of the IE and pp65 positive untreated and treated HCMV infected cells by IF (*p < 0.05, **p < 0.01, ***p < 0.001, ****p < 0.0001). **C.** Protein levels of HCMV IE (IE1:IE72, IE2:IE86) and pp65 were detected by WB analysis in untreated and pre-treated HCMV infected cells 72hpi. **D.** Quantification of the density of IE (IE72, IE86) and pp65 bands was performed using Image Studio Lite programme. Data were presented as percent of IE72, IE86 and pp65 levels after normalization to β-actin.

To assess the potential effect of Pol I inhibition on HCMV replication, MRC5 fibroblasts were pre-treated for 1.5h with the specific Pol I inhibitor CX-5461 (0.1μΜ or 1μΜ,), 5nM ActD (control for inhibition of Pol I activity) or the anti-CMV compound GCV (1mM) or left untreated. The cells were thereafter infected with HCMV (MOI 1). At 48 hours post infection (hpi), cells were collected and stained for IE and pp65 proteins. We found that both IE and pp65 protein expression were significantly lower in cells with reduced Pol I activity (Figure 1A and 1B). The strongest inhibition of IE protein expression was observed in ActD treated cells (5 nM). Reduced IE72 and pp65 protein expression was also observed by WB analysis in CX-5461 as well as in GCV treated cells at 72hpi (Figure 1C and 1D). However, IE86 protein expression was not affected at this time point by CX-5461 treatment and ActD treatment did not reduce CMV IE or pp65 protein expression levels as assessed by WB analysis at this time point (Figure 1D). ActD treated cells exhibited strongly reduced UBF staining, while only a mild effect on UBF staining was observed in CX-5461 treated cells (Figure 1A), which imply that these compounds have different effects on rRNA production in the nucleolus.

**Figure 2:**
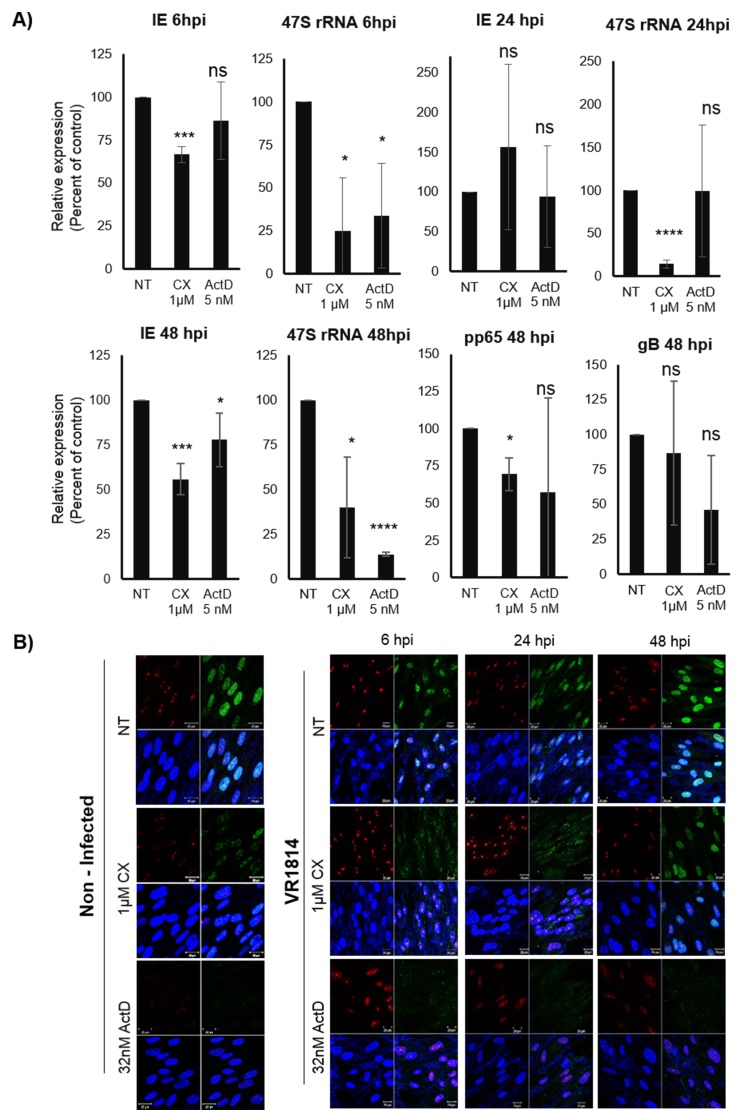
**A.** Effect of Pol I inhibition by CX-5461 on the HCMV IE (IE1:UL123, IE2:UL122), pp65 (UL83) and gB (UL55) transcripts in MRC5 HCMV infected cells. Cells were pre-treated for 1.5h with 1μΜ CX-5461 or 5nM ActD and then infected with HCMV (VR1814) for 6h, 24h and 48h. ActD was the control drug for Pol I activity. 47S was used as control for Pol I activity. Beta 2-microglobulin (B2M) was the endogenous control. Bars represent mean±SD (*n* = 3) (*p < 0.05, **p < 0.01, ***p < 0.001) **B.** Cells were stained for 5-FUrd incorporation using anti-BrdU antibody in order to confirm the inhibitory effect of CX-5461 on rRNA production. Nucleus was stained with DAPI and Fibrillarin was used as nucleolar marker. Representative immunofluorescence images are shown. Fibrillarin (red), BrdU (green), DAPI (blue).

To confirm that ActD and CX-5461 treatment reduced Pol I transcription, we next assessed the expression of the Pol I transcript 47S. At 6hpi both ActD and CX-5461 treatment significantly decreased 47S transcripts in HCMV infected cells (Figure 2A). In CX-5461 treated cells, IE transcripts were also significantly reduced (Figure 2A). At 24hpi, CX-5461 treated cells exhibited significantly reduced 47S transcript levels, which were not affected by ActD (Figure 2A). At this time point Pol I inhibition had no effect on IE transcript levels. At 48hpi, CX-5461 also significantly reduced 47S transcript levels, HCMV IE, pp65 but not gB transcript levels (Figure 2A). ActD however, only reduced IE transcripts, but did not affect pp65 or gB transcripts. At this time point the transcript levels of 47S were significantly reduced in both CX-5461 and ActD treated cells.

To further confirm that CX-5461 had an inhibitory effect on rRNA production, we examined 5-FUrd incorporation in the nucleolus with a BrdU specific antibody both in non-infected and infected cells. Fibrillarin was used as a nucleolar marker. We found that CX-5461 as well as the control ActD strongly reduced 5-FUrd incorporation in the nucleolus (Figure 2B), which suggest that CX-5461 inhibited rRNA production.

**Figure 3:**
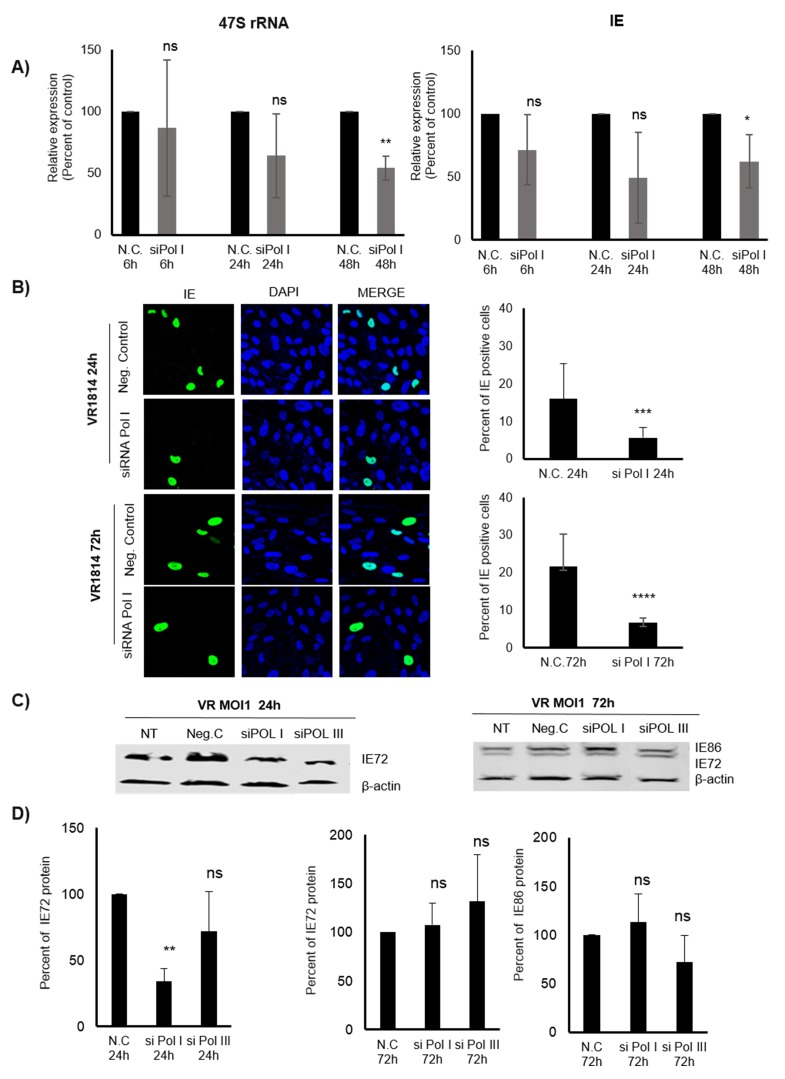
Inhibition of Pol I by siRNA affects 47S and IE (transcripts and proteins) in HCMV infected MRC5 cells **A.** Relative expression of IE transcripts in HCMV infected cells transfected with negative control or specific siRNA targeting Pol I for 6h, 24h, 48h determined by qPCR. B2M was used as endogenous control. **B.** Representative images of transfected (with negative control and siRNA targeting Pol I) and HCMV infected cells for 24h and 72h. Quantification of IE positive HCMV infected cells. **C.** Protein levels of IE (IE72 and IE86) in transfected, HCMV infected cells were determined by WB analysis and D) quantified using Image Studio Lite programme. *p < 0.05, **p < 0.01, ***p < 0.001, ****P < 0.0001

Although CX-5461 is known to specifically inhibit Pol I function, this drug may have off targets effects that could affect HCMV transcription as well. It was recently suggested that CX-5461 induces p53-independent cell cycle checkpoints mediated by ATM/ATR signalling in the absence of DNA damage [31]. To further assess a specific role of Pol I in early transcription of IE genes, we utilized siRNA to reduce Pol I function. siRNA to Pol I significantly reduced 47S transcript levels at 48 hours post treatment and infection, but not at 6 and 24 hours (Figure 3A). This could be explained by recent observations that knocking down Pol I by greater than 90% after 48 hours of siRNA transfection only reduced 47S rRNA precursor levels by 25% and 50% compared with control at 12 hours and 48 hours of transfection, respectively [31]. At 48hpi, when 47S transcript levels were low, the IE transcript levels were also significantly reduced (Figure 3A). siRNA to Pol I reduced IE protein levels already at 24hpi and maintained significantly lower levels of IE protein expression at 72hpi (Figure 3B). By WB analyses, we only observed significantly reduced IE protein levels at 24hpi with siRNA to Pol I (Figure 3C and 3D). There was no effect by siRNA to Pol I on IE72 or IE86 protein levels at 72hpi by WB (Figure 3C and 3D). siRNA to Pol III did not affect IE72 or IE86 protein levels at either of these time points (Figure 3C and 3D).

**Figure 4:**
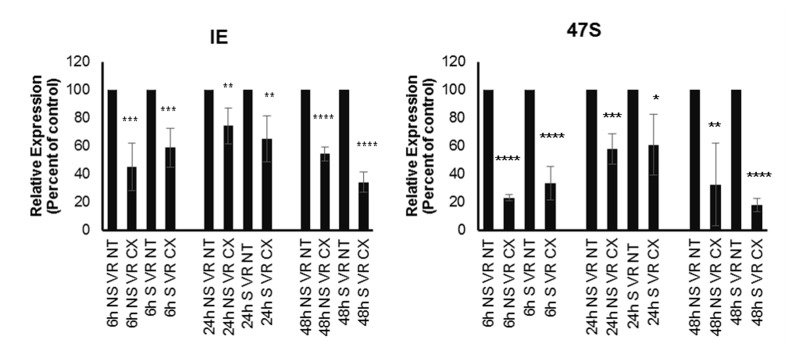
Percent of relative expression of IE and 47S transcripts in non treated treated, non synchronized and synchronized HCMV infected MRC5 cells 6h, 24h, 48hpi *p < 0.05, **p < 0.01, ***p < 0.001, ****P < 0.0001

The results were unexpected as it has been demonstrated that Pol II is the main RNA Polymerase for HCMV IE transcription. α-Amanitin is known to inhibit RNA Pol II and III without affecting Pol I activity at concentrations below 100μg. Pol-II is highly sensitive to 1-5µg/ml α-amanitin and Pol-III is sensitive to 10µg/ml α-amanitin. To assess the contribution of Pol II and Pol III activity on early transcription of HCMV, fibroblasts were treated with α-amanitin in low (1μΜ) or high (10μΜ) concentrations, or with CX-5461 (1μΜ) with or without a combination α-amanitin (1μΜ) and then infected with HCMV. Cells were collected at 6h, 24h, 48h and HCMV IE transcript levels were analysed by TaqMan PCR. We found that both low and high concentrations of α-amanitin almost completely blocked IE transcription (Supplementary Figure 1A). In these experiments, CX-5461 treatment only trended to reduce IE transcript levels at these time points, but the effect was not statistically significant (Supplementary Figure 1A). Low concentration of α-amanitin also reduced IE protein levels by WB, both in the presence or absence of CX-5461 (Supplementary Figure 1B). CX-5461 treatment did not affect IE protein expression at 72hpi by WB, which is expected as Pol II should act at this time point (Supplementary Figure 1B). As it is well known that Pol I activity fluctuates with the cell cycle, we suspected that the variability of the effect of CX-5461-treatment observed in our present experiments may be dependent on the stage of cell cycle transition. To further address this possibility, we performed a new set of experiments on cells synchronized in G0 by serum starvation and released into the cell cycle by addition of serum-containing medium. We found that under such conditions, CX-5461 treatment significantly inhibited IE transcript levels at all time points investigated (Figure 4), but less pronounced at 24 hours post infection. When analysing the cell cycle status at the indicated time points, there was no major difference between CX-5461 treated and non-treated cells at 6h (most of the cells were in G1), but we observed a shift of more cells in G2/M at 24 hours in CX-5461 treated non-infected cells (mean 7,7% in G2/M phase in non-synchronised/non-treated/uninfected cells *versus* 23% in CX-5461 treated/non-infected/non-synchronised cells, Supplementary Figure 2). These observations extend published data on the ability of CX-5461 to arrest cells in G2/M phase (31). In synchronised cells, we observed a distinct pattern: at 6h the drug drives more cells to the G2/M phase (16.4% *versus* 5.7%). In infected cells, we observed that more cells trended to be in G1 phase both in non-synchronised/CX-5461 treated and synchronised/CX-5461 treated cells at 6h and 24h as compared to 48h, where we observed a trend for increased proportion of cells in S/G2/M phases in infected/synchronised and CX-5461 treated cells. These observations suggest that the infection delays the CX-5461 mediated accumulation of cells in S/G2/M phases (see comparison of CX-5461 treated/non-infected and CX-treated HCMV infected cells (R3+R4)). Taken together, these results imply that HCMV engages Pol I for transcription only during certain phases of the cell cycle, a phenomenon that deserves further studies.

### Inhibition of Pol I does not affect HCMV IE protein expression in infected HUVEC

**Figure 5:**
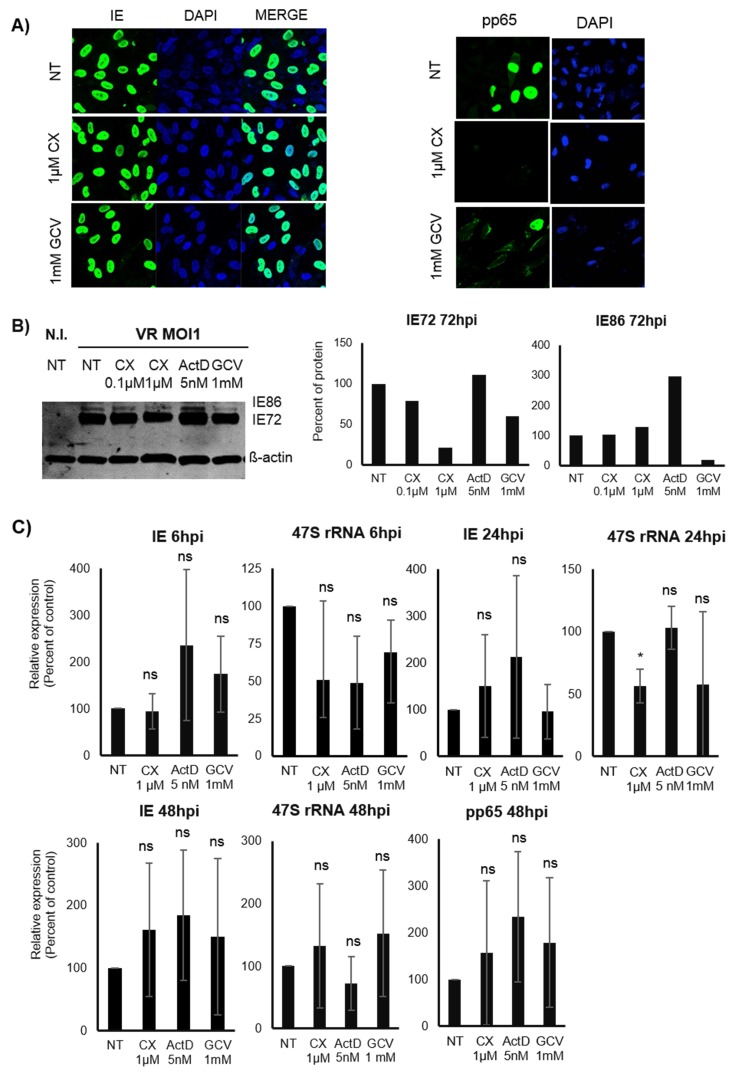
Effect of Pol I inhibition by CX-5461 on the HCMV IE and pp65 proteins and transcripts in untreated and pre-treated HCMV infected HUVEC cells Protein analysis was performed by **A.** IF staining (IE in first column and pp65 in second column: green and Dapi: blue) and **B.** by WB analysis. **C.** Percent of relative expression of IE and pp65 transcripts in pre-treated HCMV infected cells. 47S was the control for CX-5461 activity. The percent of each graph is the average percentage of three independent experiments. Bars represent mean±SD, *p < 0.05. NI: Non-infected, NT: Non-treated

HCMV infection proceeds with different sets of HCMV protein expression profiles in fibroblasts *versus* endothelial cells. We therefore next assessed whether Pol I inhibition also reduced HCMV transcript levels and protein expression in human umbilical cord endothelial cells (HUVEC). We observed that CX-5461 treatment did not affect IE protein by IF (Figure 5A, right panel) or transcript levels (Figure 5C) in HCMV infected HUVEC, but there is a reduction of pp65 (Figure 5A, left panel). However, by WB we observed reduced IE72 protein levels but not IE86 (Figure 5B). When analysing 47S transcript levels, we found that CX-5461 reduced 47S at 24hpi, but not at 6 or 48 hours (Figure 5C). Thus, CX-5461 appears to have different effects on HCMV transcription in endothelial cells and fibroblasts.

### Early inhibition of IE transcriptional activity is compensated for by Pol II and does not affect virus production in CX-5461 treated fibroblasts

**Figure 6:**
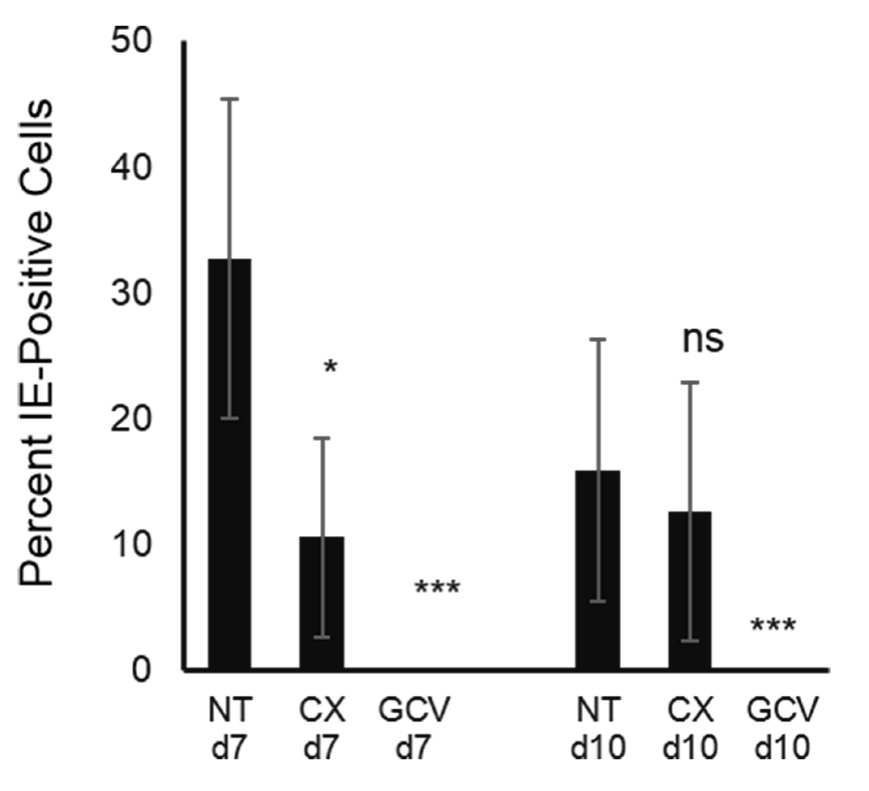
Production of infectious HCMV is not affected by Pol I inhibition at late time post infection The picture depicts percent of IE positive cells after transferring supernatants from treated HCMV infected cells to MRC5.

Our results from the experiments utilizing α-amanitin, confirm previous observations demonstrating that Pol II is the main polymerase being utilized by HCMV to produce its mRNAs [32]. However, we also found that Pol I was engaged for IE transcription, but when this occurs, appears to be dependent on the cell cycle. To assess whether the reduced levels of IE transcription and IE protein production mediated by compromised Pol I function also affected virus production, we assessed production of extracellular virus in CX-5461 or GCV treated HCMV infected cells (VR1814 MOI 1). Virus containing supernatants were collected from HCMV infected fibroblasts treated with CX-5461 or GCV at 7 and 10 dpi, transferred to new fibroblast cultures and assessed three days later for HCMV IE positive cells as a measurement of viable virus in the supernatant. CX-5461 treatment resulted in reduced virus production at 7 but not 10 dpi (Figure 6). As expected, GCV completely blocked virus, production at both 7 and 10 dpi (Figure 6). These observations suggest that Pol II later fully compensated for the Pol II-related IE transcript inhibition in the early phase of infection, in terms of resulting production of the virus.

### Pol I inhibition reduces early transcription of HSV-1 in infected MRC5 cells

**Figure 7:**
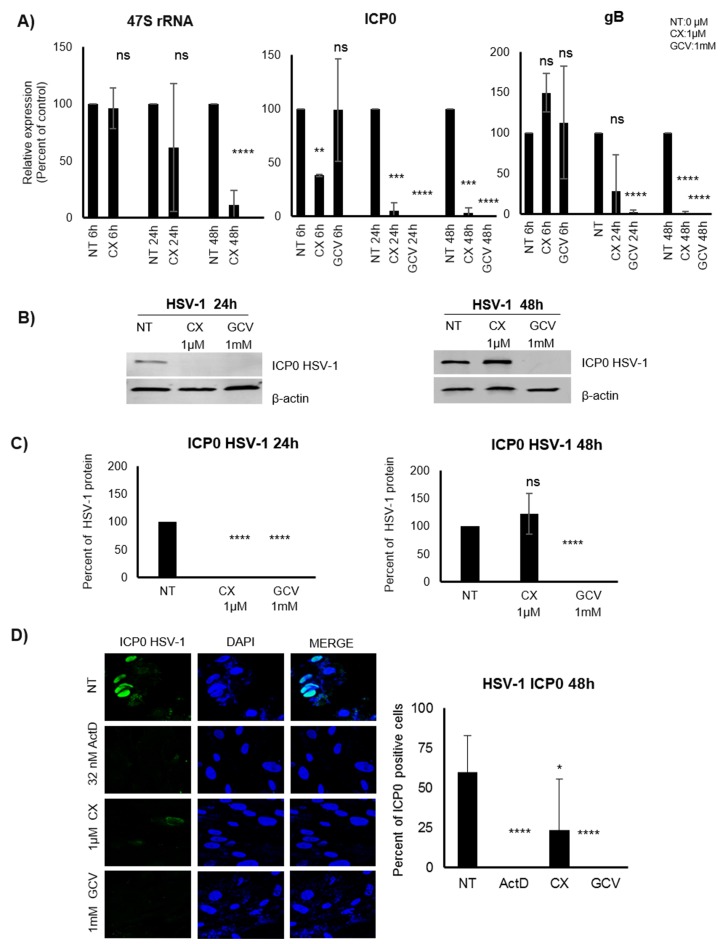
Effect of the Pol I inhibition by CX-5461 on HSV-1 infected MRC5 cells **A.** Percent of relative expression of immediate early (ICP0) and gB transcripts of HSV-1 infected (6h, 24h, 48h) cells pre-treated for 1.5h with 1μΜ CX-5461 or 1mM GCV. 47S transcript was used as control of the Pol I inhibitory effect of CX-5461. **B. C.** Infected cell protein 0 (ICP0) levels in pre-treated HSV-1 infected cells, were detected by WB (quantitative analysis of three independent experiments) and **D.** IF staining (IE: green, Dapi: blue).

We found a striking effect of Pol I inhibition of HCMV transcript levels in fibroblasts at early times after infection. To assess whether the utilization of Pol I for early transcription was unique to HCMV or is also involved in transcription of other herpes viruses, we infected CX-5461 treated MRC5 cells with HSV-1. We found that CX-5461 treatment significantly reduced levels of the HSV-1 IE transcript ICP0 at 6, 24 and 48hpi, even before we observed significantly reduced 47S transcript levels (Figure 7A). When analyzing protein levels, the ICP0 protein was not detectable by WB at 24 hours post infection in CX-5461 treated cells, but was not different as compared with non-treated cells at 48hpi (Figure 7B and 7C). Immunofluorescence staining of treated and HSV-1 infected cells showed an effect of CX-5461 (Figure 7D). Both ActD (5nM that will inhibit Pol I activity) and CX-5461 treatment, significantly reduced the number of ICP0 positive cells at 48hpi (Figure 7D). GCV treatment that was used as a control also completely inhibited HSV-1 infection at this time point (Figure 7).

### CX-5461 treatment does not affect IE transcription in MCMV infected 3T3 cells

**Figure 8:**
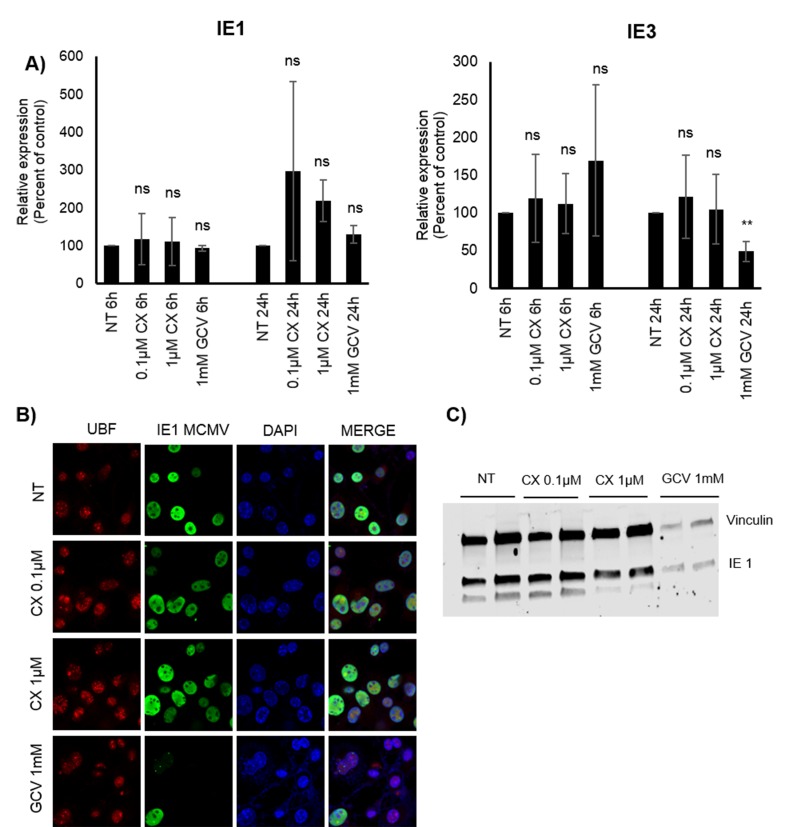
Effect of CX-5461 on MCMV infected 3T3 cells **A.** Relative expression (%) of IE1 and IE3 MCMV transcripts 6h, 24hpi. Bars represent mean±SD (*n* = 3). **B.** Representative images of immunofluorescent staining for MCMV IE1 (green) in untreated and pre-treated with 0.1 μM or 1μM CX-5461 or 1mM GCV MCMV infected 3T3 cells 48hpi. Nucleus was stained with DAPI (blue) UBF and as nucleolar marker was used (red). **C.** Protein levels of MCMV IE1 were determined also by WB at 48hpi. Vinculin was used as loading control.

To further assess whether Pol I is also being utilized by MCMV infection early in the transcription phase, we treated MCMV infected 3T3 cells with CX-5461. We did not observe any difference in IE1 or IE3 transcript levels in CX-5461 *versus* non-treated cells (Figure 8A). We also did not observe any differences in number of IE1 positive cells or total IE protein levels by immunofluorescence staining or WB (Figure 8B and 8C). GCV reduced both the number of MCMV IE1 positive cells as well as total protein levels (Figure 8B and 8C), although we did not observe any differences in transcript levels (Figure 8A) in untreated *versus* GCV treated cells at 6 or 24hpi. Thus, Pol I inhibition had no effect on MCMV IE transcript or protein production.

## DISCUSSION

Replication of HCMV is strictly controlled and the virus utilizes host cell factors for its own survival. Earlier studies have demonstrated that HCMV utilizes RNA Pol II to transcribe viral genes and produce viral mRNAs [33]. Previous studies have also shown that HCMV can specifically target the nucleolus to facilitate viral transcription and translation by mechanisms that are not well understood. As Pol I is the specific active polymerase in the nucleolus, and inhibition of rRNA synthesis results in exit of HCMV IE72 and pp65 from the nucleolus and a strong inhibition of HCMV infection [28], we hypothesized that Pol I may play a role in early viral transcription. Consistent with such hypothesis here we found that depletion of Pol I with siRNA or chemical inhibition of Pol I resulted in significantly decreased IE and pp65 mRNA and protein levels in human fibroblasts. Notably, these effects were cell-type dependent, as analogous inhibition of Pol I did not alter transcription of early HCMV genes in human endothelial cells. Furthermore, the reduced IE transcription and protein production that was seen in fibroblasts upon Pol I inhibition appeared to be cell cycle dependent. The reduced IE protein levels observed upon Pol I inhibition in the early phase of infection delayed virus production but this was compensated for at later stages of the HCMV replication cycle and did not significantly affect virus production in fibroblasts when examined at day 10 post infection. Analogous to the scenario seen in fibroblasts infected with HCMV Pol I inhibition also reduced transcription of another Herpes virus - HSV-1, in human fibroblasts. In contrast, inhibition of Pol I failed to affect MCMV transcription tested in murine fibroblasts. Taken together, these observations suggest that the utilization of Pol I by HCMV is cell type dependent and that Pol II produces viral mRNA during virus replication.

As Pol I inhibition did not affect virus production at later times after infection, we can only speculate why HCMV and HSV-1 would utilize Pol I early in the transcription phase. We demonstrate that Pol I inhibition resulted in significantly reduced IE transcript levels and IE protein production in both HCMV and HSV-1 infected cells.

Pol I transcription of ribosomal rRNA genes is confined to the nucleolus. While most of the proteins in the nucleolus are dedicated to rRNA transcription, bioinformatic analyses of the nucleolar proteome have suggested that many nucleolar resident proteins also affect mRNA maturation, chromatin structure, cell cycle control, DNA replication and repair mechanisms [34]. The nucleolus may also regulate the activity of tumor suppressors and proto-oncogenes. Since the transcriptional activity of rRNA will be rate limiting for cells under high demand for rapid growth and proliferation, it is not surprising that the Pol I machinery is targeted by many viruses including HCMV and HSV-1 to exploit its efficient transcription for their own benefit. The nucleolus plays an important role in replication of both DNA and RNA viruses [35-40], and it is also exploited by viruses to take control over the cell cycle [35, 41-46]. From the viral perspective, the role of the nucleolus in cell cycle control [19, 47] and ribosomal biogenesis is of special interest for HCMV. The HCMV protein pp65 is rapidly localised to the nucleolus after virus entry [27]. It is delivered by the virus particle and is proposed to play an important role in the early events of HCMV transcription and for the development of a lytic infection [27, 29]. During the G1 phase, rRNA synthesis and ribosomal assembly increases to meet the demands on synthesis of S phase proteins. This is regulated by nucleolar proteins, which can phosphorylate transcription factors that will interact with Pol I [28, 48-49]. Pp65 has a kinase activity [50], and may be able to phosphorylate nucleolar proteins to facilitate this process. Nucleolar accumulation of pp65 is prominent in G1 and G1/S, but very low in S or G2/M phases of the cell cycle [51]. HCMV needs to arrest in G1 phase before replication can be initiated and incoming pp65 may play a role in initiation of this process. The virus may exploit Pol I and Pol II differently in different cell types, and particularly in cells in which HCMV will establish latency in.

Pol II is, apart from being used for production of host and viral mRNAs, highly active during G1 phase and is expected to regulate the expression of host and possibly viral miRNAs. miRUL112-1 is an HCMV encoded miRNA that regulates IE expression by interacting with the IE72 RNA to reduce IE protein production. Premature expression of miRUL112-1 has been shown to decrease HCMV replication [52]. Expression of IE72 is also known to be reduced at late times of infection, which can be mediated by mirUL112-1 that is highly accumulated at late infection [53]. Induced overexpression of IE72 during acute infection also results in reduced production of virus [54]. Thus, it may be essential for HCMV to decrease IE72 expression to maintain a high replication rate. However, the role of miRUL112-1 may be different in cells in which the virus will establish latency. HCMV establishes latency in myeloid lineage cells. HCMV that enters these cells may utilize Pol I in G2/S/M phases to produce IE72 and IE86 proteins. HCMV IE86 is known to induce G1 arrest that is necessary for initiation of DNA replication [29, 55], and this may explain the high utilization of Pol II for viral mRNA production as Pol II is highly active in G1 phase. If Pol II produces miRUL112-1 that inhibits IE72 protein translation, it will result in reduced IE72 mediated activation of late gene transcription. This is expected to facilitate establishment of latency rather than promoting active virus replication. Future studies addressing these aspects would be highly interesting to further understand the molecular mechanisms regulating establishment of HCMV latency.

We found that CX-5461 inhibited both IE72 and IE86 protein expression. The effect of CX-5461 treatment appears to be more pronounced when more cells are in S/G2/M phase (Supplementary Figure 2). This reduction of IE protein expression in the early phase of infection, delayed but was not necessary for later virus replication, as virus production was reduced at 7 but not 10 dpi. It is hence possible that HCMV and the rapidly replicating HSV-1 exploit Pol I to make IE transcripts to speed up their replication at early times post infection in fibroblasts.

After 24 hours of treatment CX-5461 has been shown to arrest the cells in G2 phase [31]. This often results in accumulation of p53, which will mediate cell cycle arrest, senescence or apoptosis depending on the status of the cell [56-58]. These properties are useful for its anti-cancer effects that are currently under evaluation in clinical trials. We observed variable results on inhibition of IE transcripts by CX-5461 during the cell cycle. Depending on when treatment is initiated during the cell cycle, it may have different effects on the outcome of HCMV transcription. As CX-5461 treatment involves an effect on p53 and downstream apoptosis, cell cycle arrest and senescence, it is possible that this compound arrested cells in G2 phase, and that this also negatively influenced HCMV replication. It is also possible that the effect we observed by Pol I inhibition on HCMV transcription is affected by CX-5461s effects on other factors that interact with the Pol I machinery; i.e. Rb, p53 or Myc. Thereby Pol I could act indirectly to inhibit HCMV transcription. However, since similar results of reduced IE transcription and protein production were obtained following Pol I knockdown using siRNA, we favor a viral mechanism involving Pol I directly. Nevertheless, our results demonstrate that inhibition of Pol I affects HCMV transcription. Pol I inhibitors are presently evaluated in clinical oncology trials. Whether or not Pol I inhibition would be useful as an anti-viral strategy in CMV positive cancer forms should be interesting to evaluate further, as this virus does not replicate well in cancer cells but express IE proteins.

The effects of Pol I inhibition on production of HCMV IE transcripts and proteins were different in fibroblasts *versus* endothelial cells. In this context, it is interesting to note that replication of HCMV is faster in fibroblasts than in endothelial cells and that HCMV entry pathways are different in these two cell types. While HCMV enters fibroblasts by fusion at the plasma membrane, HCMV infects endothelial cells via receptor-mediated endocytosis (reviewed in [59]). Different cellular receptors are engaged that may also affect downstream signaling cascades. The production of IE proteins is delayed in endothelial cells. The engagement of Pol I by HCMV in fibroblasts but not endothelial cells, may therefore also depend on different cellular activation pathways induced by virus host cell interactions in different cell types.

In summary, we found that inhibition of Pol I negatively affects HCMV and HSV-1 transcription and production of IE proteins in fibroblasts but not in endothelial cells. Engagement of Pol I in the early phase of virus infection could be of benefit to speed up replication in certain cell types.

## MATERIAL AND METHODS

### Cells and viruses

Human fetal lung fibroblasts (MRC5) were purchased from ATCC and umbilical vein cells (HUVECs) were either freshly isolated from donors or purchased from Clonetics, Lonza respectively. The MRC5 were grown in minimum essential medium (MEM, Invitrogen) supplemented with glutamine, 10% fetal bovine serum (FBS) and standard Penicillin and Streptomycin (PS). The HUVEC were cultured in EBM-2 endothelial basal medium supplemented with the EGM-2 Single Quots (Clonetics, Lonza). NIH/3T3 mouse embryonic fibroblast cells were purchased from ATCC and they were cultured in Dulbecco’s Modified Eagle’s medium (DMEM, Invitrogen) supplemented with 10% fetal bovine serum (FBS) and standard Penicillin and Streptomycin (PS). MycoAlert mycoplasma detection kit (Clonetics, Lonza) was used to verify that cells were mycoplasma-free. The HCMV clinical strain VR1814 (a kind gift from Dr Giuseppe Gerna, University of Pavia, Italy), the HSV-1 (a kind offer from Prof. Maria Masucci’s lab, Karolinska Institute, Sweden), and the MCMV Smith strain (kindly provided by Dr. Frank Stassen, University of Maastricht, Netherlands) were used for the study. The VR1814 virus was propagated in HUVECs and titrated [60] in MRC5 fibroblasts (ATCC), and frozen at -80°C.

### Virus infection and treatments

Cells were pre-treated for 1.5h with either 0.1µM or 1µM CX-5461 (MedChem Express), 5nM or 32nM Actinomycin D (Sigma Aldrich), 1mM Ganciclovir (Hoffman La Roche Ltd), or were left untreated and then they were infected with HCMV strain VR1814, HSV-1 or MCMV (Smith strain). The drugs were present in the medium during the whole experiment.

### Cell cycle synchronization

For the synchronization experiments, cells in 80% confluency were starved for 48h in low-serum (0.5% FBS) culture medium. At the same time, cells were cultured at 30% confluency (non-synchronized) to avoid cell-cell contact and synchronized behaviour, in order to have them as control (growing cells). We have earlier noted that when cells are in about 70-80% confluency they exhibit a cell cycle profile as being synchronized. After 48h, the synchronized and non-synchronized cells were plated (both at approximately 30% confluency) in medium with 10% FBS. The next day (24h after plating them), they were treated for 1.5 h with CX-5461 and, thereafter infected with HCMV strain VR1814. Non-infected, infected, non-treated and CX-5461 treated cells were collected 6h, 24h, 48h post infection for IE and 47S transcript analysis and DNA content analysis.

### Analysis of cell cycle by flow cytometry

Cells were trypsinized and collected from 6-well plates. They were centrifuged 6 min 200xg at room temperature and the pellet was resuspended in PBS. Thereafter 70% ethanol was added to fix the cells and then cells were kept ≥2h on ice. The ethanol suspended cells were centrifuged for 5 min 200x g at room temperature. The pellet was resuspended in DAPI staining solution and the cell fluorescence was measured with flow Cytometer Cyan302. The analysis was done with Summit V4.3 Software.

### RNA isolation and real time PCR

Cellular RNA was isolated with the RNeasy Mini kit (Qiagen) 6h, 24h, and 48 hours post infection (hpi) and reverse transcribed to cDNA with the SuperScript III First-Strand kit (Invitrogen, Life Technologies). Real-Time PCR was performed using HCMV custom made TaqMan assays (Applied Biosystems) for detection of HCMV immediate early (IE), pp65, gB and for MCMV immediate early (IE1, IE3) primers (were designed according to [61]). TaqMan Fast universal Master Mix (2x) and Power SYBR Green Master Mix (2x) (Applied Biosystems) were used for the reactions. Power SYBR Green Master Mix was performed to detect 47S rRNA transcripts and also HSV-1 ICP0 transcripts (designed according to [62]). The human β2-microglobulin (B2M, assay ID, Hs00984230_m1) was used as a housekeeping gene for normalization. TaqMan assays were performed using a 7900HT Fast Real-Time PCR system (Applied Biosystems) with a total cycle of 40 and final volume of 10 μl per reaction. The results were analyzed with SDS 2.4 software, and the 2^-ΔΔCt^ method was used to quantify relative expression.

### Short interfering RNA (siRNA) against Pol I

For the siRNA study, HSS119452 stealth siRNA POLR1A 20nmol (Thermofisher Scientific, Invitrogen) and Lipofectamine RNAiMAX protocol (Invitrogen) following the manufacturer’s instructions, were used. AllStars Negative Control siRNA (Qiagen) served as negative control. In brief, cells at 80-90% confluency in 12-well plate were transfected with siRNA 24h before infection with HCMV at an MOI of 1. The cells were harvested for quantitative TaqMan PCR analysis 6h, 24h and 48hpi.

### FUrD

FUrd is based on the incorporation of a fluorine-conjugated uridine analogue and reflects that rRNA comprises more than 80% of cellular RNA. Cells were incubated with 2 mM FUrd (Sigma-Aldrich, cat. no: F5130) in DMEM and incubation was allowed for 10 min at 37°C. To stop, medium was removed and cells were washed with cold PBS. For detection of incorporated FUrd, cells were fixed with a 4% formaldehyde solution in PBS at room temperature, permeabilized with a 0.5% Triton X-100 solution in PBS and incubated with Monoclonal Anti-BrdU antibody clone BU-33 (Sigma Aldrich, cat. no: B8434). Actinomycin D was used as control drug.

### Immunofluorescence

Cells were seeded in culture slides and infected with HCMV strain VR1814, HSV-1 or MCMV MOI of 1. At 6h, 24h, 48hpi the slides were fixed with PFA 4% for 15 minutes and washed three times with PBS. They were incubated with Fc receptor blocker (Innovex Biosciences, cat. no: NB309) and then blocked using Dako Protein Block (Dako Cytomation, cat. no: X0909). Thereafter, the slides were stained with a monoclonal mouse anti-HCMV IE (1:500, MAB810, Merck-Millipore, cat. no: 5027-5), monoclonal mouse anti-HSV-1 ICP0 (1:1000, Virusys Corporation, cat. no: H1A027), pp65 (Novocastra, cat. no: NCL-CMVpp65), mouse anti-m123/IE1 MCMV (1:1000, Croma 101 Ab) and rabbit polyclonal UBF: Upstream Binding Factor (1:70, Santa Cruz Biotechnologies, cat. no: sc-9131). The immunoreactivity was revealed by adding secondary antibody conjugated to Alexa Fluor and analyzed with Zeiss confocal microscope LSM 700.

### Western blot analysis

Briefly, cells were lysed with RIPA buffer (supplemented with 1% protease inhibitor) at 24h, 48h and 72hpi. The concentration of the proteins was measured using Pierce TM BCA Protein Assay Kit. Samples were boiled in 1x Laemmli buffer with 5% β-Mercaptoethanol for 10 minutes. The WB assay was performed with 4-15% precast gels (Biorad) and then the samples were transferred onto PVDF membranes. The membranes were stained using, as primary antibodies, mouse polyclonal β-actin (1:1000, Santa Cruz Biotechnologies, cat. no: sc-47778), or rabbit polyclonal vinculin (1:20.000, Abcam, cat. no: ab73412) antibody, mouse anti-HCMV IE (1:1000, MAB810, Merck-Millipore, cat. no: 5027-5), mouse anti-HCMV pp65 (Novocastra, cat. no: NCL-CMVpp65), mouse monoclonal anti-HSV-1 ICP0 (1:4000, Virusys Corporation, cat. no: H1A027), mouse monoclonal anti-m123/IE1 MCMV (1:1000, Croma 101 Ab) and secondary antibodies conjugated with IRdye (Goat anti-rabbit IRDye 800CW (cat. no: P/N 925-32211), and Goat anti-mouse IRDye 680RD, cat. no: P/N 925-68070, LICOR). They were developed using Odyssey CLx (LICOR) and quantification of the band density was performed with Image Studio Lite programme.

### Viral Infectivity in cell culture supernatants

MRC5 were grown as triplicate cultures in 6-well plates to approximately 80% confluence and were pre-treated for 1.5h with either CX-5461, or GCV and subsequently infected with HCMV or left uninfected. Cells were washed at 3 dpi and re-treated with the corresponding drug. Supernatants were collected at 7 and 10 dpi and stored at -20°C. For determination of infectious virus in the supernatants, MRC5 were seeded on culture slides and were infected with 10µl supernatant. Slides were fixed at 3 dpi for immunofluorescent staining of IE (see above).

### Statistical analysis of data

Data were analysed by two-tailed t test, using Prism (version 5, GraphPad 339 Inc.). Data are mean ± SEM for 3 experiments. P-values are denoted as follows: *P < 0.05, **P < 0.01, ***P < 0.001, ****P < 0.0001.

## SUPPLEMENTARY MATERIALS FIGURES


