# Supplementary material for: Human cytomegalovirus and Herpes Simplex type I virus can engage RNA polymerase I for transcription of immediate early genes
Source: Oncotarget. 2017 Oct 29;8(57):96536–52. doi: 10.18632/oncotarget.22106 (PMC5722503; doi:10.18632/oncotarget.22106)
Supplement: Supplementary file 1 [file oncotarget-08-96536-s001.pdf]

Human cytomegalovirus and Herpes Simplex type I virus can engage RNA polymerase I for transcription of immediate early genes

SUPPLEMENTARY MATERIALS

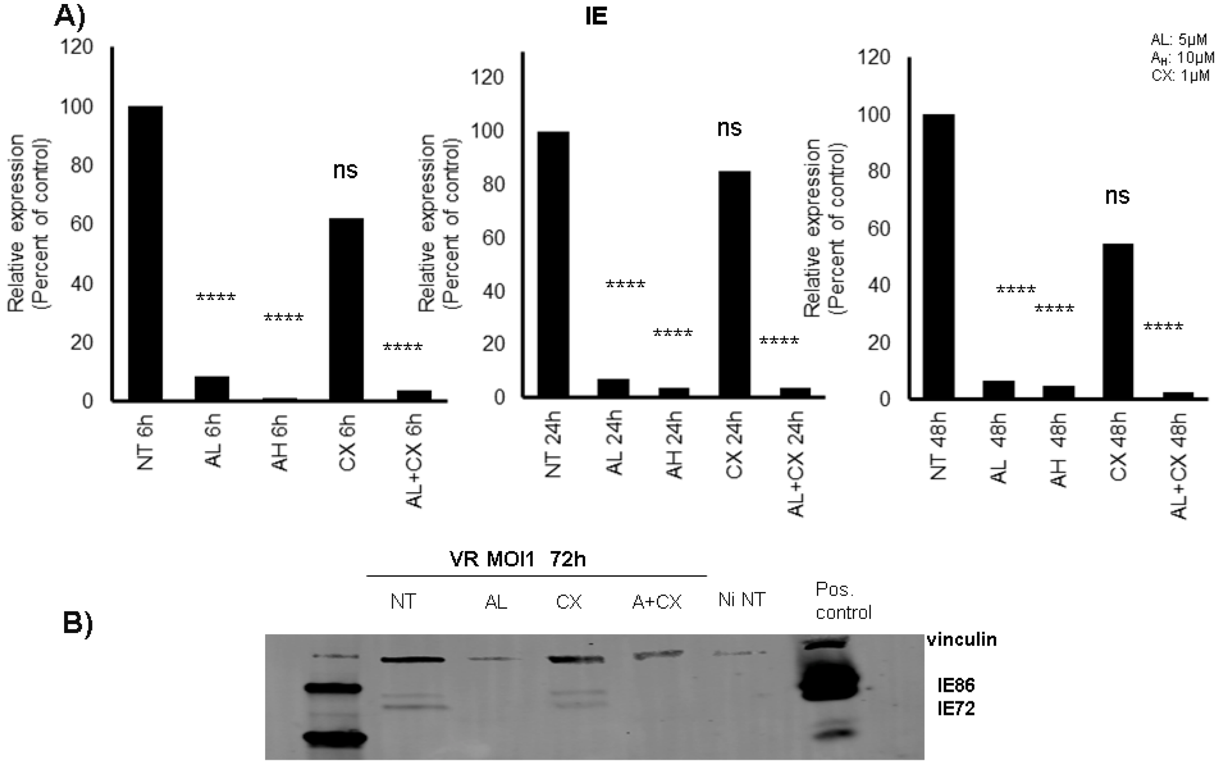

Supplementary Figure 1: A) Pol II is the main RNA Polymerase for HCMV IE transcription. α-amanitin inhibits RNA Pol II and III without affecting Pol I activity at concentrations below 100µM. Pol-II is highly sensitive to 1-5µg/ml α-amanitin and Pol-III sensitive to 10µg/ml α-amanitin. Cells were treated with α-amanitin in low (1 µM) and high (10 µM) concentration, CX-5461 in 1 µM or combination of low α-amanitin and CX-5461 then infected with HCMV for 6h, 24h, 48h. Data represent the average of percentage of three different experiments. **B**) Western blot was performed to show the IE protein levels. Vinculin was used as loading control.

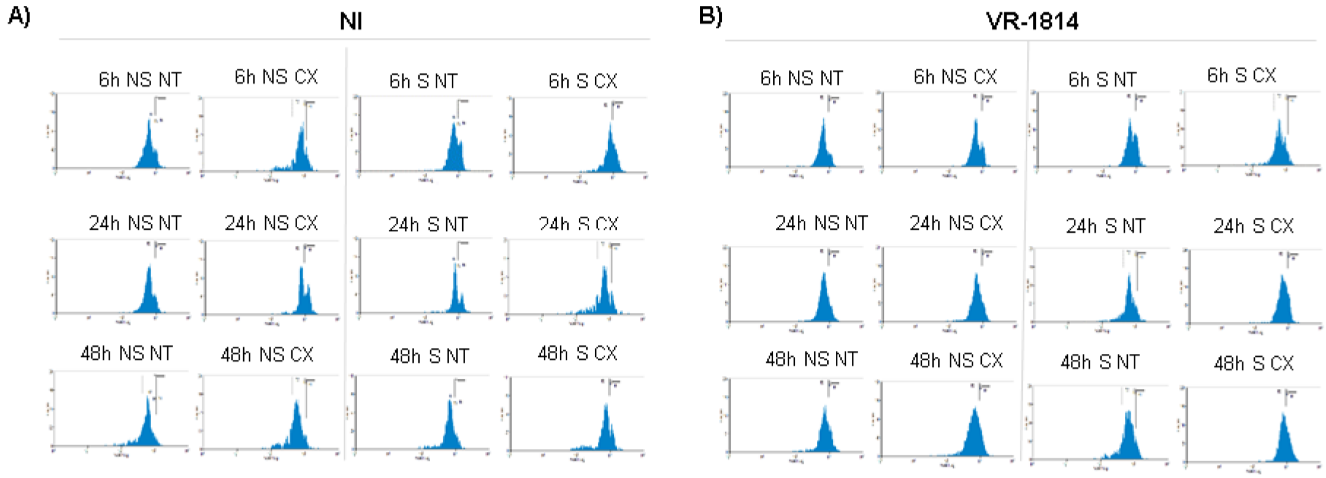

C)

	NI NS NT	NI NS CX	NI S NT	NI S CX
6h	G1:69,80<br>S:9,8<br>G2/M:6,18	G1:61,17<br>S:9,98<br>G2/M:5,74	G1:67,06<br>S:14,36<br>G2/M:9,25	G1:59,15<br>S:12,83<br>G2/M:16,36
24h	G1:71<br>S:9,98<br>G2/M:7,72	G1:56<br>S:15,46<br>G2/M:23	G1:65,28<br>S:9,76<br>G2/M:17,89	G1:63,61<br>S:13,47<br>G2/M:16,58
48h	G1:67,25<br>S:6,39<br>G2/M:4,6	G1:69,11<br>S:9,45<br>G2/M:8,94	G1:69,99<br>S:9,35<br>G2/M:7,46	G1:66,77<br>S:14,23<br>G2/M:11,38

D)

	VR NS NT	VR NS CX	VR S NT	VR S CX
6h	G1:73,24<br>S:9,84<br>G2/M:8,83	G1:69,24<br>S:11,52<br>G2/M:7,29	G1:68,17<br>S:16,39<br>G2/M:8,41	G1:69,1<br>S:13,97<br>G2/M:8,06
24h	G1:71,71<br>S:11,59<br>G2/M:9,6	G1: 68,80<br>S:11,47<br>G2/M:10,93	G1:64,31<br>S:16,8<br>G2/M:18,24	G1:71,58<br>S:12,68<br>G2/M:8,2
48h	G1:65,22<br>S:15,21<br>G2/M:13,19	G1:64,35<br>S:16,25<br>G2/M:14,65	G1:67,67<br>S:13,95<br>G2/M:13,67	G1:59,62<br>S:19,67<br>G2/M:20,66

Supplementary Figure 2: Analysis of cell cycle of non-infected non-treated and CX-5461 treated non synchronised, synchronised, and HCMV infected non-treated and CX-5461 treated non synchronised, synchronised MRC5 cells 6h, 24h and 48hpi. **A**, **B**) Representative histogram analysis of cell cycle distribution at different conditions. **C**, **D**) Percentages of the cells in the different phases. NI: Non infected, NS: non synchronised, VR: VR1814 infected cells, S: synchronised, NT: Non treated, R2:G1, R3:S, R4:G2/M. It has to be mentioned that the percent that is left in order to have 100% is the G1 sub phase.
